# “I have SMA, SMA doesn’t have me”: a qualitative snapshot into the challenges, successes, and quality of life of adolescents and young adults with SMA

**DOI:** 10.1186/s13023-021-01701-y

**Published:** 2021-02-22

**Authors:** Allison Mazzella, Mary Curry, Lisa Belter, Rosángel Cruz, Jill Jarecki

**Affiliations:** Cure SMA, 925 Busse Road, Elk Grove Village, IL 60007 USA

**Keywords:** Spinal muscular atrophy, Quality of life in SMA adolescents and young adults, Qualitative research, Emotional health, Disability, Peer-support group, Accessibility, Fatigue, Dependence

## Abstract

**Background:**

With the approval of three treatments for spinal muscular atrophy (SMA) and several promising therapies on the horizon, the SMA adolescent and young adult populations are expected to evolve in the coming years. It is imperative to understand this cohort as it exists today to provide optimal care and resources, as well as to assess possible treatment effects over time. In 2018, Cure SMA launched two initiatives geared towards understanding adolescents and young adults with SMA, ages 12–25. First, Cure SMA launched a Quality of Life (QoL) survey to capture quantitative and qualitative information on this specific age demographic. Concurrently, Cure SMA invited SMA-affected individuals, ages 12–25, to create a three-minute video on their everyday experiences living with SMA. An inductive thematic analysis of the free-text survey questions along with the video contest findings are reported here.

**Results:**

Eighty-five individuals—6 type Is, 58 type IIs, and 21 type IIIs—completed the Quality of Life free-response, while six individuals participated in the SMA awareness video contest. In both settings, individuals detailed a variety of challenges, including but not limited to forming or maintaining close relationships, experiencing feelings of isolation, challenges with accessibility, independence, and dealing with the stigma of being perceived as mentally disabled. Individuals also discussed their successes, including but not limited to higher education enrollment and attendance, development of quality friendships, and perseverance through obstacles. Additionally, notably in the survey, 39% of respondents requested the creation of an SMA peer support group in efforts to connect with each other as well as collectively navigate the aforementioned challenges they face.

**Conclusion:**

Together, these findings provide a rare glimpse into the unique mindsets, challenges and motivations of SMA adolescents and young adults, via patient-reported measures instead of caregiver proxy. The adolescent and young adult age demographics assessed represent a critical transition period in life and in SMA care. No one understands the needs of an adolescent or young adult with SMA better than the individuals themselves, and it is critical to encapsulate their insights to affect change.

## Background

Spinal muscular atrophy (SMA) is an autosomal recessive neurodegenerative disease characterized by progressive muscle weakness often resulting in loss of movement, significant disability, and premature death [1–3]. SMA is estimated to affect between 8500 and 10,300 children and adults in the US [4–6]. SMA is traditionally classified into three main subtypes based on age of symptom onset and maximal achieved motor abilities [7]. In SMA type I, individuals are diagnosed within the first 6 months of life and never achieve independent sitting. Traditionally, without treatment, these individuals require intensive supportive care [2, 8–11]. In SMA type II, (Kugelberg–Welander disease) patients experience symptom onset within the first 18 months of life and have achieved the ability to sit independently but will not achieve the ability to walk unaided. Over time, these patients may develop difficulties involving chewing and swallowing, respiration, as well as progressive scoliosis [12, 13]. In SMA type III, individuals achieve the ability to stand and walk independently but may lose these abilities over time [12, 13]. In SMA type IV, otherwise known as adult-onset SMA, symptom onset typically occurs at age 30 or later, and as such, this type was not identified within our target age population [12]. Regardless of subtype, the disease burden of SMA is substantial, and complexly impacts affected individuals and their loved ones across multiple dimensions [6, 14–17].

With the approval of three treatments as of August 2020, the phenotypic presentation and prevalence of SMA in adolescent and young adult populations is expected to change. Due to this shift, disease classification is moving away from subtype and towards demographics based on motor function. All data presented below will be displayed in accordance with motor function while information related to SMA type is used to validate.

In previous years, a variety of initiatives have qualitatively captured clinical meaningfulness and the experiences of the adult SMA population [16, 18–20]. However, to our knowledge, this study is the first to directly capture information specific to quality-of-life directly from affected adolescents (12–18) and young adults (19–25), as the adolescent perspective tends to be captured via caregiver proxy, while the young adult perspective is captured along with that of adults across various ages [5, 14, 16–20]. It is necessary to further understand the factors this population deems most important in the overall improvement of quality-of-life. A clearer understanding may provide an opportunity for researchers to develop or optimize outcome measures and resources to further support adolescents and young adults affected by SMA.

## Methods

### Quality of Life (QoL) Survey

The Quality of Life Survey consists of four open-ended questions. The objective of this survey was to provide an opportunity for adolescents and young adults affected by SMA to share, in their own words, individual experiences about living with SMA as well as highlight topics important and specific to their stage of life. The four free text questions inquired on biggest obstacles, the impact of SMA on schooling and socialization, and potential resources to improve quality-of-life. No guidelines were given towards these responses in order to help identify a variety of themes. The Quality of Life Survey was distributed as part of a larger survey which was comprised of three sections: (1) demographics information, (2) SMA Health Index instrument (SMA-HI, a patient reported outcome measure developed by Dr. Chad Heatwole at the University of Rochester) (Heatwole [15, 18, 21] abstract), and (3) free text response. The SMA-HI information collected will be presented in a future publication.

The survey was generated through Survey Monkey and approved by the Western Institutional Review Board (Approval Number 1-11240001-1). Written informed consent was obtained online from all adult respondents prior to the conduct of any research related activities. For minor adolescent respondents (ages 12–18), written consent was obtained online from a parent/guardian, and assent was obtained from the survey respondent thereafter.

The survey was advertised through targeted email blasts sent to over 400 families using Cure SMA’s membership database as well as Cure SMA’s social media platforms. Cure SMA is a non-profit patient advocacy organization for SMA, which currently maintains a robust database that features data from over 8000 individuals affected with SMA [6]. As of June 1, 2018, 412 individuals between the ages of 12–25 were contacted within the database. Eighty-three percent of this cohort is composed of self-reported type II and III affected individuals [6]. Confidence intervals were calculated to provide power for each comparison. Sample size calculations, however, were not done prior to the study because open-ended responses were evaluated here and therefore, effect size could not be assessed using prior studies. Based on an expected response rate of about 20%, we expected about 80 responses that would elicit a 95% confidence level of and a 9.8% margin of error. Email blasts and social media reminders were disseminated biweekly. Materials directed at consenting adults contained a direct link to the survey, while materials designed for families of minors contained information regarding the parent/caregiver consenting and the participant assenting process prior to the survey. All consents and assents were collected electronically. The survey remained open from November 2018 through January 2019 for a total of 8 weeks—an initial 6-week submission period with a 2-week extension.

An inductive thematic approach was adopted for qualitative analysis of the free text collected in the survey. All questions were evaluated for themes independently of one another. For each question, all responses were manually sorted by the primary author into themes through the identification of key words and sentiments. Key words were identified retroactively of survey completion, for purposes of data analyses. Although the prompts were open-ended, there was a high concordance in responses across participants, particularly regarding the final question. Once all responses were categorized, they were sorted by age, SMA type, and respondent’s maximum motor function at time of diagnosis and time of survey completion, with all demographic information being self-identified. Maximum motor function status was defined as; non-independent sitter (head control, maintain seated position supported), sitter/non-independent walker (maintain seated position unsupported, crawl, cruise), and independent walker (walk independently). Statistical significance based on response age and maximum motor function at time of survey completion was assessed using Fischer’s exact test. For detailed information regarding key words and phrases included in each theme as well as statistical significance results, please see Additional file 1: Tables S1–S4.

### SMA awareness video contest

The objective of the SMA awareness video contest was to raise awareness about the impact/burden of SMA on adolescents and young adults, and learn about the strategies they use/have developed to help navigate their day to day life and the challenges that come with managing this multifaceted and complex disease. Contest guidelines included general topic suggestions that participants could discuss within a 3-min time limit. Topics suggestions included but were not limited to schooling, everyday routine, relationships with family and friends, and activities/interests. Post-completion, this project was submitted for and received IRB exemption for the purposes of retrospective analysis (Approval Number 1-1305771-1). For more information on contest guidelines, please review Appendix 1.

The contest was announced on Cure SMA’s website and social media platforms in October 2018 and ran through January 2019. Targeted e-mail blasts featuring contest guidelines were disseminated to over 400 families of SMA on a biweekly basis, using the Cure SMA membership database; recruitment reminders were also posted on Cure SMA’s web and social media pages. The contest submission period was 6 weeks in duration, after which point all videos were reviewed and approved by Cure SMA staff for video length and use of appropriate language, and subsequently uploaded to Cure SMA’s YouTube channel. All submitters received an Amazon gift card, as a thank-you for their participation. The three videos with the most "likes" received an additional incentive. Video submissions are still accessible; a full link along with contest results can be found in Appendix 2. Video submissions were analyzed for key content themes as well as percentage of time spent on each theme.

In the results detailed below, individuals involved with the survey will be referred to as ‘respondents’, while individuals involved with the video contest will be referred to as ‘participants’. All individuals quoted will be identified by their age, gender, and motor function level at the time of completion, and all quotes cited are directly their words.

## Results

### Quality of life survey

#### Participation rates and sample characteristics

The QoL survey elicited eighty-five (85) responses, representing a 21.3% response rate, with a 95% confidence level and a 9.4% margin of error. Of the respondents, forty-four (44) were adolescent minors (ages 12–18, mean age 15), while forty-one (41) were young adults (ages 19–25, mean age 21). Fifty-three (53) individuals identified as female, thirty-one (31) as male, and one as non-binary. The most prevalent maximum motor function level at both time of diagnosis and at time of survey completion was sitter/non-independent walker. As respondents were recruited from the survey through the Cure SMA database, demographic information could be verified. Sample demographics and clinical characteristics can be found on Table 1**.**

**Table 1:** Quality of life survey demographics

	Total age cohort	Adolescents (12–18)	Young adults (19–24)
Total	85	44 (52%)	41 (48%)
SMA type			
Type I	6 (7%)	4 (5%)	2 (2%)
Type II	56 (66%)	28 (33%)	28 (33%)
Type III	23 (27%)	12 (14%)	11 (13%)
Gender			
Female	53 (62%)	28 (33%)	25 (29%)
Male	31 (37%)	15 (18%)	16 (19%)
Non-binary	1 (1%)	1 (1%)	0
Motor function at time of diagnosis^a^			
Non-independent sitter	25 (29%)	13 (15%)	12 (14%)
Sitter/non-independent walker	37 (44%)	20 (24%)	17 (20%)
Walker	23 (27%)	11 (13%)	12 (14%)
Motor function at time of survey completion*			
Non-independent sitter	31 (37%)	17 (20%)	14 (17%)
Sitter/non-independent walker	40 (47%)	19 (22%)	21 (25%)
Walker	14 (16%)	8 (9%)	6 (7%)

85 individuals responded to the QoL survey. Non-binary was included as a gender option, and describes individuals who identify with no one gender

^a^Motor function abilities were divided into three categories: non-independent sitter (head control, maintain seated position supported), sitter/non-independent walker (maintain seated position unsupported, crawl, cruise), and walkers (walk independently). All demographic information was self-identified. All percentages are based on the total surveyed population of 85 respondents

##### What is the most difficult aspect of balancing SMA symptoms with everyday life?

The first free text question of the QoL survey addressed the most difficult aspect of balancing SMA symptoms with the everyday lives of adolescents and young adults (Table 2, Additional file 1: Table S1). The highest cited factor overall was dependence on others and the lack of independence. One respondent wrote, “At this point I don’t know any different, but it’s the fact that you always have to rely on someone that starts to become overwhelming” (Respondent #63; sitter, age 22, female). The respondents who indicated dependency were most likely to be sitter/non-walker (p = 0.008).

**Table 2:** Responses to QoL free text question 1

What is the most difficult aspect of balancing your SMA/SMA symptoms with everyday life?							
	Age			Motor function			
	Adolescents n = 44% (95% CI)	Young Adults n = 41% (95% CI)	p value	Non independent sitter n = 31% (95% CI)	Sitter/non-independent walker n = 40% (95% CI)	Independent walker n = 14% (95% CI)	p value
Accessibility	15.91 (6.64–30.07)	14.63 (5.57–29.17)	0.87	**0**	**20 (9.05–35.65)**	**35.71 (12.76–64.86)**	0.002*
Advocating	2.27 (0.06–12.02)	2.44 (0.06–12.9)	1	3.23 (0.08–16.70)	0	7.14 (0.18–33.9)	0.147
Balance	6.82 (1.43–18.66)	7.32 (1.54–19.92)	1	3.23 (0.08–16.70)	12.5 (4.2–26.8)	0	0.256
Dependence	22.73 (11.47–37.84)	24.39 (12.36–40.30)	0.857	**16.13 (5.45–33.73)**	**37.5 (22.73–54.20)**	**0**	**0.008^**
Everyday	9.09 (2.53–21.67)	9.76 (2.72–23.13)	1	9.68 (2.04–25.75)	5 (0.61–16.92)	21.43 (4.66–50.80)	0.151
Fatigue	6.82 (1.43–18.66)	17.07 (7.15–32.06)	0.186	19.35 (7.45–37.47)	7.5 (1.57–20.39)	7.14 (0.18–33.9)	0.258
Finding care	**0**	**9.76 (2.72–23.13)**	0.05*	9.68 (2.04–25.75)	2.5 (0.06–13.16)	0	0.374
Mental health	6.82 (1.43–18.66)	0	0.242	6.45 (0.79–21.42)	0	7.14 (0.18–33.9)	0.181
other	6.82 (1.43–18.66)	0	0.242	3.23 (0.08–16.7)	2.5 (0.06–13.16)	7.14 (0.18–33.9)	0.567
Pain	9.09 (2.53–21.67)	7.32 (1.54–19.92)	1	12.9 (3.63–29.83)	7.5 (1.57–20.39)	0	0.451
Social	13.64 (5.17–27.35)	7.32 (1.54–19.92)	0.486	16.13 (5.45–33.73)	5 (0.61–16.92)	14.29 (1.78–42.81)	0.25

Dependence and the lack of independence was the highest-cited difficult aspect, incurring 20 total respondents. Percentages are out of total number of respondents (n = 85). The table values represent statistically significant differences in responses between either the age groups (2 bins) or between the motor function groups (3 bins) using a chi square or Fisher’s exact test. Highlighted findings were found to have statistical significance; asterisk(*) indicates significance by Fisher’s exact test and carrots(^) indicates significance by Chi2 test

Other highly cited factors included accessibility, fatigue, and social concerns. Regarding accessibility, one respondent wrote, “There is an awful lot of planning that has to go into getting around a huge campus like where I go to school. I worry all the time about stupid things like whether or not my chair lift in my car will work, whether it will snow outside by building and I will slip on the ice, what happens if I fall in the shower, will my smart drive be charged to get me through the day” (Respondent #86, walker, age 20, female). There was a statistically significant difference in discussing accessibility by motor function (p = 0.005), as the majority of individuals reporting accessibility had a maximum motor function of independent walker. Fatigue was most frequently mentioned by individuals with a maximum motor function status of sitter/non-independent walker than an independent walker. One respondent remarked, “Everything is difficult because depending on the day, my muscles get tired which makes me tired” (Respondent 30, non-independent sitter, age 12, female). Regarding socialization, one respondent wrote, “It’s hard to always keep up with my friends” (Respondent 20, sitter, age 14, non-binary).

Other factors mentioned with less frequency included the burden of pain and physical symptoms, finding proper care and finding a healthy life balance. Multiple non-independent sitting individuals cited pain as their most difficult aspect of balancing life, one writing, “Just dealing with the pain.” (Respondent 01, non-independent sitter, age 15, male). There was a statistically significant difference between adolescents and young adults citing finding one-on-one care to be the most difficult aspect of balancing life, with only young adults citing this factor (p < 0.05). Responses are categorized by age and motor function at the time of survey completion in Table 2 and the themes by type in Additional file 1: Table S1.

#### How does SMA affect schooling?

The second question of the QoL free text inquired about the impact of SMA and SMA symptoms on schooling (Table 3, Additional file 1: Table S2). The largest response category was no effect with 13 responses. Some respondents simply indicated “it doesn’t”, while others elaborated on their situations. “SMA does not affect my schooling. I am currently enrolled in college and taking 14 units on campus*”* (Respondent 36, sitter, age 18, male). This response was primarily indicated by individuals with a maximum motor function of non-independent sitter or sitter/non-independent walker.

**Table 3:** QoL free text responses to question 2

How does SMA affect your schooling?							
	Age			Motor function			
	Adolescents n = 44% (95% CI)	Young Adults n = 41% (95% CI)	p value	Non independent sitter n = 31% (95% CI)	Sitter/non-independent walker n = 40% (95% CI)	Independent walker n = 14% (95% CI)	p value
Accessibility	11.36 (3.79–24.56)	9.76 (2.72–23.13)	0.55	6.45 (0.79–21.42)	7.5 (1.57–20.39)	28.57 (8.39–58.10)	0.077
Activity limitation	13.64 (5.17–27.35)	2.44 (0.06–12.9)	0.11	3.23 (0.08–16.70)	15 (5.71–29.84)	0	0.163
Aid	9.09 (2.53–21.67)	12.2 (4.08–26.20)	0.733	12.9 (3.63–29.83)	12.5 (4.2–26.8)	0	0.457
Everyday	11.36 (3.79–24.56)	4.88 (0.60–16.53)	0.435	9.68 (2.04–25.75)	2.5 (0.06–13.16)	21.43 (4.66–50.80)	0.073
Fatigue	**2.27 (0.06–12.02)**	**17.07 (7.15–32.06)**	0.026*	6.45 (0.79–21.42)	12.5 (4.2–26.8)	7.14 (0.18–33.9)	0.79
Homeschool	**18.18 (8.19–32.71)**	**0**	0.006*	6.45 (0.79–21.42)	12.5 (4.2–26.8)	7.14 (0.18–33.9)	0.79
Keep up	6.82 (1.43–18.66)	9.76 (2.72–23.13)	0.707	9.68 (2.04–25.75)	7.5 (1.57–20.39)	7.14 (0.18–33.9)	1
More effort	4.55 (0.56–15.47)	14.63 (5.57–29.17)	0.147	16.13 (5.45–33.73)	7.5 (1.57–20.39)	0	0.255
No effect	15.91 (6.64–30.07)	14.63 (5.57–29.17)	0.87	16.13 (5.45–33.73)	17.5 (7.34–32.78)	7.14 (0.18–33.9)	0.642
Other	4.55 (0.56–15.47)	7.32 (1.54–19.9)	0.669	9.68 (2.04–25.75)	2.5 (0.06–13.16)	7.14 (0.18–33.9)	0.381
Writing	2.27 (0.06–12.02)	7.32 (1.54–19.9)	0.349	3.23 (0.08–16.70)	2.5 (0.06–13.16)	14.29 (1.78–42.81)	0.217

Although the most common response was ‘no effect’ with 13 total participants, responses were distributed evenly across a variety of factors including accessibility, aid, and fatigue. When categorized by motor function at time of survey completion, sitters were the main respondents of ‘no effect’, while non-sitters more commonly detailed accessibility, aid, and time/effort challenges. Percentages are out of total number of respondents (n = 85). The table values represent statistically significant differences in responses between either the age groups (2 bins) or between the motor function groups (3 bins) using a chi square or Fisher’s exact test. Highlighted findings were found to have statistical significance; asterisk (*) indicates significance by Fisher’s exact test and carrots (^) indicates significance by Chi2 test

Conversely, the other most frequent responses indicated much higher burden. These answers include accessibility, aid and assistance, homeschooling, and fatigue. Accessibility received 9 responses, mainly comprised of individuals with various levels of motor function, but who identified as Type III. One respondent wrote, “I use an elevator and a rolling backpack, and the other students do not” (Respondent 72, walker, age 12, male). Aid and assistance also received 9 responses. One respondent wrote, “It forces me to require a 1 on 1 aide” (Respondent 05, non-independent sitter, age 18, male). 8 individuals discussed homeschooling, for reasons including germ exposure and placement struggles. This response was primarily indicated by adolescents and is statistically significant by age groups (p = 0.006). A respondent detailed, “I stopped going to public school, now I do it at home. They put me with all of the other special needs kids because I was in a wheelchair” (Respondent 64, sitter, age 14, female). Fatigue was also discussed by 8 individuals, this time primarily indicated by young adults. This finding is also statistically significant (p = 0.026) by age groups. One respondent stated, “Because of my lack of energy, I could not handle being a full-time student” (Respondent 74, SMA-III, age 23, female). Although these individuals have faced academic challenges, many of them report being highly successful. Of the young adult survey respondents, 16 are currently enrolled in college, with an additional 12 individuals having graduated college and 3 individuals holding a graduate degree. Responses are categorized by age and motor function at the time of survey completion in Table 3 and the themes by type in Additional file 1: Table S2.

#### How does SMA affect socialization?

The third free-text question asked how SMA affects socialization (Table 4, Additional file 1: Table S3). The highest responses were accessibility and no effect, each detailed by 16 respondents. Accessibility respondents once again primarily consisted of individuals with the maximum motor function of independent walker. One respondent remarked, “Since I use a wheelchair now, I have to think about accessibility. Are my friends’ homes accessible for me? Are the locations of social activities accessible? I definitely won’t have the same amount of independence as a sixteen-year-old who doesn’t have SMA” (Respondent 72, sitter, age 16, male).

**Table 4:** QoL free text responses to question 3

How does SMA affect your social life?							
	Age			Motor function			
	Adolescents n = 44% (95% CI)	Young Adults n = 41% (95% CI)	p value	Non independent sitter n = 31% (95% CI)	Sitter/non-independent walker n = 40% (95% CI)	Independent walker n = 14% (95% CI)	p value
Accessibility	11.36 (3.79–24.56)	26.83 (14.22–42.94)	0.068	16.13 (5.45–33.73)	22.5 (10.84–38.45)	14.29 (1.78–42.81)	0.708
Activity limitation	18.18 (8.19–32.71)	14.63 (5.57–29.17)	0.659	12.9 (3.63–29.83)	15 (5.71–29.84)	28.57 (8.39–58.10)	0.423
Communication difficulties	4.55 (0.56–15.47)	9.76 (2.72–23.13)	0.349	12.9 (3.63–29.83)	5 (0.61–16.92)	0	0.312
Dating	2.27 (0.06–12.02)	2.44 (0.06–12.9)	1	0	5 (0.61–16.92)	0	0.653
Dependence	6.82 (1.43–18.66)	7.32 (1.54–19.9)	1	**0**	15 (5.71–29.84)	**0**	0.04*
Feeling judged	11.36 (3.79–24.56)	0	0.056	3.23 (0.08–16.70)	7.5 (1.57–20.39)	7.14 (0.18–33.9)	0.705
Misunderstood	6.82 (1.43–18.66)	12.2 (4.08–26.20)	0.474	19.35 (7.45–37.47)	2.5 (0.06–13.16)	7.14 (0.18–33.9)	0.056
No effect	27.27 (14.96–42.79)	9.76 (2.72–23.13)	0.053	19.35 (7.45–37.47)	15 (5.71–29.84)	28.57 (8.39–58.10)	0.533
No social life	9.09 (2.53–21.67)	4.88 (0.60–16.53)	0.677	12.9 (3.63–29.83)	2.5 (0.06–13.16)	7.14 (0.18–33.9)	0.209
Other	2.27 (0.06–12.02)	12.2 (4.08–26.20)	0.102	3.23 (0.08–16.70)	10 (2.79–23.66)	7.14 (0.18–33.9)	0.542

‘No effect’ on socialization was most commonly cited by non-independent sitter, though it was followed closely by major effects including accessibility concerns and an absence of socialization. Percentages are out of total number of respondents (n = 85). The table values represent statistically significant differences in responses between either the age groups (2 bins) or between the motor function groups (3 bins) using a chi square or Fisher’s exact test. Highlighted findings were found to have statistical significance; asterisk (*) indicates significance by Fisher’s exact test and carrots (^) indicates significance by Chi2 test

A high rate of respondents indicated no effect, incurring responses from all levels of motor function. One respondent stated, “It does not really affect my social life because my friends are cool” (Respondent 06, non-sitter, age 14, female). Conversely, some survey respondents felt the opposite, with little to no socialization. “What social life?” one respondent remarked (Respondent 03, non-sitter, age 15, male). Another respondent wrote, “SMA affects my social life very much. I never want to go out because everyone stares at me everywhere I go” (Respondent 19, non-sitter, age 12, female). This answer was discussed by 6 respondents of all motor function levels.

Other factors impacting socialization include activity limitations, communication difficulties, and feeling judged or misunderstood. While describing activity limitations, one respondent wrote, “I am not able to do many activities. It affects my social life and my love life (by that I mean I don’t have one)” (Respondent 85, walker, age 22, female). Additionally, one respondent noted communication difficulties, stating, “People have a hard time understanding me” (Respondent 11, non-sitter, age 20, male). Regarding feelings of judgement, one participant wrote, “Many adults and teens speak to me as though I’m much younger or will talk to the person I’m with acting as though I’m not really there” (Respondent 47, sitter, age 18, female). Dependence as a response in this section once again obtained statistical significance by motor function categories, with all respondents being sitter/non-independent walker (p = 0.04) rather than independent walker. Responses are categorized by age and motor function at the time of survey completion in Table 4 and the themes by type in Additional file 1: Table S3, respectively.

#### What resources should exist for teens and young adults with SMA?

The final question of the QoL free text asked respondents what resources they would like to see created for this specific SMA population (Table 5, Additional file 1: Table S4). Most often mentioned, 33 responses, across all ages and levels of motor function, suggested the creation of peer support groups. One respondent elaborated, “A group where we can just talk to each other about the issues we have, how we deal with them, joke with each other, and just connect over it” (Respondent 44, sitter, age 18, male). *A*nother respondent remarked, “Support groups to help cope with the emotional and mental toll of SMA” (Respondent 53, sitter, age 22, female). Separate, but closely related, was the idea of a big/little buddy support program. One respondent wrote, “It would be useful to pair young adults with older individuals with SMA, like a buddy to learn from” (Respondent 13, non-sitter, age 23, female).

**Table 5:** QoL free text responses to question 4

What resources do you think should exist for teens and young adults with SMA?							
	Age			Motor function			
	Adolescents n = 44% (95% CI)	Young Adults n = 41% (95% CI)	p value	Non independent sitter n = 31% (95% CI)	Sitter/non-independent walker n = 40% (95% CI)	Independent walker n = 14% (95% CI)	p value
A Cure	9.09 (2.53–21.67)	0	0.117	0	5 (0.61–16.92)	14.29 (1.78–42.81)	0.116
Accessible activities	6.82 (1.43–18.66)	0	0.242	6.45 (0.79–21.42)	0	7.14 (0.18–33.9)	0.181
Accessible transit	0	7.32 (1.54–19.92)	0.108	6.45 (0.79–21.42)	2.5 (0.06–13.16)	0	0.755
Big/little support	6.82 (1.43–18.66)	2.44 (0.06–12.9)	0.617	3.23 (0.08–16.70)	7.5 (1.57–20.39)	0	0.654
Caregivers	0	7.32 (1.54–19.92)	0.108	6.45 (0.79–21.42)	2.5 (0.06–13.16)	0	0.755
College/independence materials	**0**	**100.00%**	0*	12.9 (3.63–29.83)	12.5 (4.2–26.8)	14.29 (1.78–42.81)	1
Equipment/technology	18.18 (8.19–32.71)	4.88 (0.60–16.53)	0.057	6.45 (0.79–21.42)	15 (5.71–29.84)	14.29 (1.78–42.81)	0.591
Other	2.27 (0.06–12.02)	4.88 (0.60–16.53)	0.607	3.23 (0.08–16.70)	5 (0.61–16.92)	0	1
Support groups	40.91 (26.34–56.75)	36.59 (22.12–53.06)	0.683	38.71 (21.85–57.81)	42.5 (27.04–59.11)	28.57 (8.39–58.10)	0.655
Unknown	6.82 (1.43–18.66)	9.76 (2.72–23.13)	0.707	9.68 (2.04–25.75)	5 (0.61–16.92)	14.29 (1.78–42.81)	0.516
Video games	9.09 (2.53–21.67)	0	0.117	6.45 (0.79–21.42)	2.5 (0.06–13.16)	7.14 (0.18–33.9)	0.502

The overwhelming majority response to Question 4 was peer-support groups. Question 4 exhibited the largest group consensus. Percentages are out of total number of respondents (n = 85). The table values represent statistically significant differences in responses between either the age groups (2 bins) or between the motor function groups (3 bins) using a chi square or Fisher’s exact test. Highlighted findings were found to have statistical significance; asterisk (*) indicates significance by Fisher’s exact test and carrots (^) indicate significance by Chi2 test

Another frequent suggestion was the creation of college and independent living transition materials. This response incurred 11 responses of all motor function levels. One respondent stated, “More info on going to college independently; as an adult I have met others with SMA who were surprised I went to college and lived independently” (Respondent 52, sitter, age 25, female). Other ideas included improved access to equipment and technology, accessible transportation maps and activities, and accessible video games. Regarding improved access to equipment and technology, one respondent wrote, “Help with better access. Help to get a van for using power chair away from home and school. Grants for families” (Respondent 69, sitter, age 18, female). Regarding accessible activities, respondents suggested the creation of a wheelchair accessible waterpark, video game console and sport specifically modified to fit the needs of all SMA severities. Lastly, a few respondents wrote that a cure would be an excellent resource. Responses are categorized by age and motor function at the time of survey completion in Table 5 and the themes by type in Additional file 1: Table S4.

### Awareness video contest

The video contest received six submissions: four from adolescent minors and two from young adults. Five of the six individuals identified as type II; the remaining participant was a young adult with type III. Ambulatory status was assessed from the video submissions. Please see Table 6 for full demographic information.

**Table 6:** Video contest demographics

Awareness video contest demographics				
	Age	Gender	Type	Ambulatory status
Respondent 1	15	Female	II	Non-ambulant
Respondent 2	15	Female	II	Non-ambulant
Respondent 3	17	Female	II	Non-ambulant
Respondent 4	22	Female	III	Ambulant
Respondent 5	20	Female	II	Non-ambulant
Respondent 6	17	Male	II	Non-ambulant

6 individuals participated in the video contest

Although the video contents had a much smaller sample size, the findings align with those of the QoL survey. Video content was divided into 6 subthemes discussed by all participants: social engagement, everyday life, physical health, emotional health, other obstacles and overcoming obstacles (Table 6). Of note, all video submissions discussed social engagement in detail, with participants spending a minimum of 20% of their submission time on the topic. Participants highlighted the importance of friendships, as well as the importance of family members, pets, paras and other key relationships. Regarding the impact of SMA on socialization, once again participants spoke to both ends of the spectrum and the high level of impact it had on their emotional well-being. Once participant said, ““I have a few really close friends, and they’re absolutely my best friends. I think that we’re closer than someone who wasn’t in a wheelchair because they walk through SMA with me” (Participant 01, SMA-II, age 15, female). Conversely, another discussed, “I realized that not being included was much harder than it seemed to be. It’s not that I was bullied, but that no one wanted me to be their friend.” (Participant 06, SMA-II, age 17, male).

Submissions highlighted fatigue and feeling unable to participate in everyday life as difficult aspects of managing SMA. One participant detailed the difficulty of transitioning from a fully-independent walker to needing assistive support. “It’s really hard to get up and down stairs, to bring my walker places, to need friends to wait or slow down. I never had to think about any of it before. It’s a challenge I overcome, but I did not think it would be so soon” (Participant 04, SMA-III, age 22, female). Additionally, participants spoke to both ends of the spectrum while describing the impact of SMA on schooling. One participant noted, “I needed an aid as well as many other accommodations, which resulted in a fair amount of arguments with people who just don’t understand” (Participant 03, SMA-II, age 18, female) while another detailed, “I don’t want to be known as the girl in the wheelchair, I want to be known as this cool teenage girl. I’m really into student government, yearbook, French club, Humane society…” (Participant 02, SMA-II age 15, female). While the video submissions did not discuss specific resource recommendations, an inspiration for quite a few participants to create videos was to be a role model for struggling members of the Cure SMA and disabled adolescent communities. One participant said, “I hope to be an inspiration to all the kids out there that grew up like me, who are in wheelchairs and have self-doubt.” (Participant 05, SMA-II, age 20, female).

One element of the video contest that was not covered in the QoL survey is overcoming adversity. Each video participant highlighted challenges they experienced while also discussing their motivations and strategies of resilience. One participant said, “I keep a blog and make YouTube videos to show you can have a disability and still have a life that’s full and fulfilling. I hope to be an inspiration to all the kids out there that grew up like me, who are in wheelchairs and have self-doubt.” (Participant 05, age 20, female). Another participant detailed, “My family and I, we love to get out and travel. We don’t let inaccessible routes or people tell us ‘You can’t go that way.’ Usually, we figure out a way to do it. There are challenges, but there are ways to help, that’s what SMA is all about” (Participant 01, SMA-II, age 15, female). For more information on the topics discussed in the video contest, please refer to Table 7**.**

**Table 7:** Video contest topic breakdown

Awareness video contest topic breakdown (n = 6)			
Theme	% of participants addressing theme	Subtheme	Sample quote
Social engagement			
	100	Friendship	“My friends treat me the same way they treat anyone else, that’s why we’re best friends”
	100	Key relationship (family, pet, helper)	“My brother is a runner and he runs for me”
	50	Lack of social engagement	“No one invited me to be their friend”
Everyday life			
	100	Schooling	“With school, I have to have a full-time para”
	100	Activities	“One of my passions is the arts, I like to paint”
	83.3	Everyday routine	“I still do chores, I still help out around the house”
Physical health			
	100%	Mobility	“A wheelchair can’t go that way”
	100%	Overall disease burden	“I have all the physical demands of being disabled”
Emotional health			
	50	Frustration	“It’s hard to go upstairs, and I get mad at myself sometimes when I can’t do it”
	33.3	Depression	“Realizing how my peers treated me, depression overcame me”
Other obstacles			
	100	Dependence	“I have to have someone do everything for me”
	83.3	Being labeled/treated “disabled”	“Stop with these misconceptions that everyone who is disabled is mentally slow or just sitting around”
Overcoming obstacles			
	100	Defying other’s expectations	“Just because you can’t physically do something doesn’t mean you can’t follow your dreams”
	100	Accepting + adapting	“I’ve learned to adapt and teach myself to write with both hands”

Contest participants discussed various topics regarding life as adolescents and young adults living with SMA. A sample of direct quotes from each topic is shown here

### Impact on mental health

Across the platforms, mental health was discussed by twelve different survey respondents. When detailing difficult obstacles, one survey respondent elaborated, “I was just diagnosed with depression and anxiety mostly from the constant worrying about my life and having SMA, I think that in some cases having SMA makes me want to work harder and prove others wrong, but at the same time there are a lot of days where I just want to give up and say what's the point” (Respondent 86, walker, age 20, female). Other respondents were more succinct but equally impactful. One respondent wrote in the difficult obstacles question, “Depression. It makes everything worse and less bearable” (Respondent 09, SMA-II, age 16, female). Survey respondents also discussed the emotional impact of being considered disabled simply by having a wheelchair. In the socialization question, one respondent explained, “I feel like people judge me just by seeing the wheelchair. People treat me like I’m a baby or mentally disabled or too innocent and I’m none of those things!” (Respondent 61, sitter, age 19, female). Another respondent wrote, “It makes me sad when people stare at me. I know they’re probably just ‘curious’, but still, it makes me upset” (Respondent 18, non-sitter, age 18, female).

In the awareness video contest, mental health issues were discussed in-depth by two participants. One participant elaborated, “After losing my aide of three years and realizing how I was being treated by my peers, depression overcame me. I could no longer stay well” (Participant 03, SMA-II, age 18, female). Another said, “As soon as middle school started, I was thrown into a deep depression, and I still have not fully recovered…It is the physical demands of being disabled that would eventually shape my personality to being an introvert.” (Participant 06, SMA-II, age 17, male).

### Successes and accomplishments

Although not directly addressed in either incitive, it is evident that many adolescents and young adults affected by SMA are leading productive lives despite challenges. In the schooling section of the QoL survey, a few individuals highlighted their academic accomplishments. One survey respondent wrote, “Graduated from the Wharton School at the University of Pennsylvania” (Respondent 29, sitter, age 23, male) while another wrote, “I get As and Bs” (Respondent 06, non-sitter, age 14, female). Many survey respondents also emphasized their interests outside of their disease, such as video games, swimming and wheelchair sports. In response to the question regarding socialization, one respondent wrote, “I still go dancing!!!!!” (Respondent 68, sitter, age 23, female).

Similar successes were also emphasized in the awareness video contest. One video contest participant highlighted their future plans. “After I finish my degree, I plan on working with children who are similar to me, hopefully inspiring them to do what they want to do with their lives. Just because you can’t physically do something doesn’t mean that you can’t follow your dreams” (Participant 05, SMA-II, age 20, female). All contest participants detailed their interests, ranging from clubs to YouTube, attending concerts, and traveling. Additionally, many participants highlighted unique experiences. One participant reflected, “If you want to live twenty hours away from your parents, go for it. If you want to go hunting for [sic] your dad, do it. If you want to go skydiving, why not? My only advice is, don’t be afraid of a challenge. Be excited for it” (Participant 06, age 17, male).

## Discussion

In comparing our population to the SMA adult population captured in previous publications, our top responses coincide with their key findings. Both accessibility and fatigue were reported as significant in multiple questions. Accessibility was a high response in our study in relation to schooling and socialization. This correlates with the identification of accessibility and limited mobility as symptoms with the greatest impact on SMA adults in the SMA Patient-Reported Impact of Symptoms (PRISM-SMA) study (n = 359) [18]. Fatigue is also often identified as clinically meaningful, and it is highlighted both in the above study as well as in our findings in relation to greatest challenge and schooling. Furthermore, dependence and the lack of independence was found to be a difficult challenge in our study in correlation to motor function, and the existing literature. Achieving greater independence with treatment was considered to be extremely significant, and therefore, clinically meaningful to parents/caregivers as well as affected adults [8, 14]. Similarly, results from a global polling with SMA parents/caregivers and affected adults at an externally led Patient-Focused Drug Development Meeting corroborated the finding that any treatment that increases independence is acutely meaningful [17].

### Impact of age and motor function

When breaking our population into cohorts of adolescents (ages 12–18) and young adults (ages 19–25), it is evident that certain themes are most relevant to certain age groups versus others. Only young adults requested the creation of college and independent transition materials and young adults also more frequently reported fatigue issues in school and issues finding care. Conversely, adolescents were more likely to report being homeschooled. With these differences noted between age groups, majority of the key factors impacting the day to day lives of adolescents and young adults were largely shared by both groups.

There was a statistically significant difference between maximum motor function and identifying accessibility and dependence as the most difficult aspect of balancing SMA; accessibility concerns were mainly voiced by participants who previously walked independently or were able to walk independently at the time of survey completion. While accessibility was a main issue for these individuals, the sentiment was not shared by those with a maximum motor function of non-independent sitters, who more frequently detailed pain or communication difficulties. Non-walkers were the most likely to voice challenges regarding dependence or reliance on others, and these results were significant with respect to socialization and considered a difficult aspect of disease management. While motor function status was correlated with some responses, many responses had no correlation. At least one individual of every motor function group discussed difficulties regarding mental health, feeling left out, or needing more time and effort in school. Additionally, the no effect responses detailed in schooling and socialization all incurred varied responses from all levels of motor function.

### No effect vs severe effect of SMA on schooling and socialization

In both the schooling and socialization questions of the free text, a parabola effect was observed in high frequency answers. A similar number of respondents felt that either, SMA greatly affected, or had no effect on their schooling or socialization, two significant aspects of life in this age period. The majority of those who felt that SMA did not impact schooling did not overlap with individuals who felt that SMA did not impact their socialization. It is important to note that neither of these questions received a large group consensus unlike the categories of everyday life or resources, with similar frequencies reported amongst many responses.

### Impact on mental health

Although mental health was not the most frequently cited factor in any category, the impact of SMA on mental health can be severe [16, 17, 19, 20]. Similar concerns regarding mental health have been observed in both the adult SMA population as well as the larger adolescent population. It is estimated that approximately one in five adolescents struggles with mental health or a diagnosable mental health disorder [22]. Additionally, in a poll conducted among caretakers and individuals with SMA, during the SMA Patient Focused Drug Development Meeting with the FDA, 105 adults with SMA reported having experienced anxiety (79%), depression (61%), and social isolation (63.8%) as a result of coping with SMA and SMA symptoms [17]. Furthermore, in a series of 25 interviews conducted with SMA-affected young adults and adults in Australia, mental health was highlighted as a major unmet need, particularly in stressful times related to SMA symptoms including loss of function, social isolation, stigma and questions of self-worth [20]. While this impact was not found to affect all individuals, those who were affected described being severely impacted, noting effects ranging from withdrawal from mainstream school to ongoing battles with depression. Within our survey, amongst those respondents that indicated mental health concerns, there were no similarities in responses to other questions.

The specific psychosocial and developmental challenges observed in our research are similar to those found in the larger adolescent and young adult populations. These population archetypes struggle with feeling left out at school, navigating adulthood, and finding independence [22]. Our results indicate that SMA affected adolescents and young adults experience typical age-demographic challenges compounded by the burden of coping with and managing a significant disability.

### Overcoming adversity

As discussed in the corresponding results section, most of the SMA adolescent and young population surveyed are leading successful and productive lives. The resilient spirit exhibited in our findings complements existing publications in which adversity, resilience and grit were found to be key components of adolescence and young adulthood with SMA affected individuals. Study participants described strategies including focusing on abilities rather than disabilities, and prioritizing self-worth and value [19].

### Potential impact of peer support groups

A peer support group was the most requested and most desired resource to support this population. Such a resource is not uncommon for disease populations. In a July 2013 publication from Patient Education and Counseling, researchers found that while there were benefits to both mentor/mentee programs and age-matched support groups, the latter had the greatest potential for symmetrical and mutually beneficial relationships leading to greater emotional support [23]. This is consistent with our findings, although a mentoring buddy program was requested, the requests for a peer support program were much greater.

Furthermore, peer support groups are common resources for this age demographic within and outside of the rare disease community. In a 2015 study regarding cystinosis support groups for emerging adults (n = 49), participants reported valuing the connection and support they received from those who understand their lived experience [24]. Additionally, in a 2018 study involving a literature review regarding 15 studies on the impact of peer support groups on adolescents struggling with mental health, support groups were shown to be positive influences in the management of short-term and long-term problems [22]. These articles are consistent with our population’s interest and demonstrate the strong potential for benefit were this resource to be created. Undoubtedly, teens and young adults with SMA view peer support groups as preferred mechanism to cope with the reported isolation they experience as a result of their disease.

### Study limitations

There are several important limitations to note with our research. Although the size of our population is a strength, there is a low participation of type I individuals. Traditionally, most of those affected with SMA type I do not survive past the age of two and those who do survive present with a very severe phenotype (requiring respiratory and feeding support and little to no movement) that would make direct participation in these studies very challenging. Furthermore, there is a greater number of females than males in our study. This limitation makes it difficult to summarize our findings to the greater SMA population, as it does not represent the entire population. Additionally, because this was a qualitative research study using open ended responses, rather than a validated patient reported outcome measures with prior usage in the SMA population, predications about possible differences in responses between sub-populations were not feasible, making a priori power calculations impossible. Instead, confidence intervals were calculated when comparing sub-populations to provide indication of statistical precision. Furthermore, as the survey is directly patient-reported, there may have been mis-reported data on SMA type and other demographic information. Some information that could impact results, such as mental health status, economic status, and motor function gain/loss was not collected. Lastly, the awareness video contest response rate is hypothesized to be much lower than the survey response rate due to the high demands of the contest design, including creating and editing a video submission, publicly posting to YouTube, and the subsequent contest campaigning. Although the small cohort is not intended to represent the entire SMA population, their anecdotes and insights are intended to supplement a fuller picture of this age cohort and largely align with the findings of the larger QoL free response findings.

### Implications and future directions

The findings of this research corroborate previous findings on disease burden and quality of life in adults and caregivers with SMA [14–16, 19]. These findings also point to gaps and tangible opportunities to guide patient organizations and other key SMA stakeholders to generate important community and school resources that may help to improve the experience of teens and young adults living with SMA. For instance, the consensus and enthusiasm for a peer support group was unmatched and demonstrates a clear need in this community. Equally salient is the creation of college and independent living transition materials and information about accessibility in schools and college campuses. Additionally, these studies provide a perspective captured for the first time specifically on teens and young adults with SMA, along key dimensions that impact the quality-of-life in this population, at a unique point in time with the recent approval by the FDA of therapies for SMA. There is much more to be explored, both regarding the current generation as well as future adolescents receiving transitional care in the landscape of SMA’s changing phenotypes. Future awareness efforts are needed to combat the stigma that comes with a disability, and the barriers regarding accessibility, to ensure this population fully experiences the best life they possibly can. Lastly, developing programs that support optimal functioning and mental health among teenagers and young adults living with a complex disease is of utmost importance.

## Conclusion

As SMA treatments and care expand, more and more SMA-affected children will live to become teenagers and young adults with bright futures ahead. It is imperative to understand the current life experience in order to improve the quality-of-life for this and future generations. It is evident that this population has a big voice with valuable insights, and no one understands the needs of the SMA community better than those directly affected. Despite all of the challenges and complexities that come with living with SMA as adolescents and young adults, these teens are living full and successful lives. Indeed, via this work, we have learned that these individuals have SMA but SMA does not have them.

### Supplementary information

**Additional file 1**. contains Supplementary Tables I-IV, denoting the quality of life survey questions' key words and response breakdown by SMA type.

## Data Availability

Additional data is available from the authors upon request.

## Appendix 1: Cure SMA teen video contest guidelines

Cure SMA is hosting a video contest to raise SMA awareness and provide the opportunity for teens and young adults (aged 12–21) to share their experiences. We want you to participate!!

The purpose of this contest is to raise awareness about the impact that SMA has on teens and young adults, and the strategies you use to help navigate your day to day life. Your voice is crucial. Your video will help to increase awareness of the disease. We hope that your peers can learn from you!

Who can enter?

- You must have a diagnosis of Spinal Muscular Atrophy (SMA)
- You must be 12–21 years old
- You must live in the United States
- You may work individually or as a group (up to 3 individuals), but one person must apply and represent on behalf of the group. This individual must have a prior diagnosis of SMA
- Parents and adults can be advisors, but we respectfully request the teen/young adult be the primary driver
- Parental permission is required for contestants under 18 years old
- Immediate family and staff of Cure SMA are ineligible to submit

How to enter?

- We want to know more about all of the creative techniques you utilize to remain an active teenager or young adult. Create a short (3 min or less) video that highlights the following:

1. How does SMA impact your goals as a teenager/young adult as you deal with the issues that others your age face including (but not limited to):
  - School/work
  - Friendships
  - Independence
  - Dating

In addition to item #1 above, all videos must also include one or more of the following topics.

2. What care/treatment options are the most effective for you as you manage your symptoms? (physical therapy, hydrotherapy, massage therapy, breathing assistance, nutrition etc.) Please refrain from mentioning any drug name or clinical trial\; videos including this information will be disqualified from the contest.
3. Even with the most well thought out plan, life gets in the way! What challenges do you face along the way and how do you manage?
4. In addition to treatment, do you have additional strategies that contribute to your daily joy/improved quality of life? (dog companion, helper, devices, treatments, etc.)

- Upon completion, please submit your video, via email, to CREATE EMAIL ADDRESS. All videos will be reviewed prior to Cure SMA staff posting the content to the Cure SMA’s YouTube® account. Participants under the age of 18 years of age must have parental consent. A signed consent form along with a copy of identification is required with each submission; those over the age of 18 must also consent and provide a copy of identification. Please contact Mary Curry at mary.curry@curesma.org or 847.264.1189. Notification of acceptance will be provided via email along with a direct link to the uploaded content.
- Individuals or groups may submit more than one video as long as each video covers different topics. Please see above for the list of subject areas.
- Winners will be chosen based on total “Likes” obtained by each video; every “Like” equals one vote. All participants are encouraged to share their video with friends and family members to increase the total number of votes received. Only videos submitted to Cure SMA and uploaded to Cure SMA’s YouTube® account will be considered.
- Make sure you read and approve the terms and conditions. Easy!!

Exclusion criteria.

- All entrants 18 years of age and older are required to submit a signed consent and photo ID via email to Mary Curry at mary.curry@curesma.org; entrants under the age of 18 year of age are required to obtain permission from all parents and/or legal guardians as well as a copy of a photo ID. Those that do not comply are excluded from participation.
- All videos will be reviewed for content prior to Cure SMA’s acceptance of video entry. All videos that do not feature the required subjects will be disqualified. Any videos that mention any drug name or clinical trial will be disqualified. The participant will be notified via email if disqualified.

Timeline is as follows:

Submission Period: October 15, 2018 to November 20, 2018.

Cure SMA Video Upload Date: November 28, 2018.

Time Period to Market Your Film: November 28, 2018 to December 9, 2018.

Announcement of Finalists and Grand Prize Winner: December 21, 2018.

Prizes:

Winners will be chosen based on the total “Likes” obtained on their video between November 28, 2018 at 12:00 am CST to December 9, 2018 11:59 pm CST.

Prizes will be awarded as follows:

1st Place: Amazon Gift Card for $150.

2nd Place: Amazon Gift Card for $125.

3rd Place: Amazon Gift Card for $100.

Winners will be notified by e-mail.

Terms and Conditions.

For optimal video upload guidelines visit the YouTube® website.

Participants of Cure SMA’s Video Contest agree to YouTube’s® Community Guidelines (https://www.YouTube.com/yt/about/policies/#community-guidelines) and Terms of Service (https://www.YouTube.com/static?gl=US&template=terms). YouTube® is not a sponsor of your contest and all participants are required to release YouTube® from any and all liability related to this contest.

Entrants may only submit their own original works.

All submitted videos are subject to approval and may not be accepted if they are found to be offensive or in violation of copyright. This includes the use of copyrighted music, digital, movie, or printed works. See “Acceptable Site Use” in ACS Terms of Use.

Cure SMA reserves the right to use submitted videos on the Cure SMA website and in other promotions, including but not limited to the Cure SMA channel on YouTube®, Cure SMA’s SMArt Moves microsite, and the Cure SMA publications. Cure SMA and the organization’s industry partners are release from any and all liability related to this contest.

Any personal information supplied by you to Cure SMA will be subject to Cure SMA's privacy policy posted at http://www.curesma.org/privacy-policy.html. By entering the Contest, the Entrant/Parent/Legal Guardian grants Cure SMA permission to share your E-mail address and any other personally identifiable information with You Tube or with any co-sponsor solely for the purpose of administration and prize fulfillment. Cure SMA will not sell, rent, transfer or otherwise disclose your personal data to any third party other than as described above herein or in the privacy policy.

Please contact Mary Curry at mary.curry@curesma.org or 847.264.1189 with any questions.

## Appendix 2: Cure SMA teen video link and results

Cure SMA recently hosted a video contest to raise SMA awareness and provide the opportunity for teens and young adults (aged 12–21) to share their experiences. The purpose of this contest is to raise awareness about the impact that SMA has on teens and young adults, and the strategies used to help navigate day to day life.

Special thanks to everyone who participated in our contest and helped to make it a success! Thank you for sharing your journey. We continue to learn from each of your stories.

Although the contest has ended, please continue to view and share all of the contest entries on Cure SMA’s YouTube Channel at https://www.youtube.com/watch?v=u9baqO4lno8&list=PLQVcp9RApBmz3QN_CBFXGlUAWeoTLmKMn&index=2

Congratulations to all the participants and thank you to all that voted!
