# Supplementary material for: “I have SMA, SMA doesn’t have me”: a qualitative snapshot into the challenges, successes, and quality of life of adolescents and young adults with SMA
Source: Orphanet J Rare Dis. 2021 Feb 22;16:96. doi: 10.1186/s13023-021-01701-y (PMC7898731; doi:10.1186/s13023-021-01701-y)
Supplement: Supplementary file 1 — Additional file 1. contains Supplementary Tables I-IV, denoting the quality of life survey questions' key words and response breakdown by SMA type. [file 13023_2021_1701_MOESM1_ESM.docx]

**Additional file 1: Table S1. Key Words and Breakdown for Survey Question 1**

| **Question 1: What is the most difficult aspect of balancing your SMA/SMA symptoms with everyday life?** | | | | | |
| --- | --- | --- | --- | --- | --- |
| **Theme** | **Key Words** | **Total Responses** | **SMA Type** | | |
|  |  |  | **I** | **II** | **III** |
| **Dependence** | *dependent, lack of independence, reliance, rely on people* | **20** | 0 | 18 | 2 |
| **Accessibility** | *wheelchair accessible, getting around, mobility, difficulty going places* | **13** | 0 | 6 | 7 |
| **Fatigue** | *tired, limited energy, need rest, weakness* | **10** | 0 | 7 | 3 |
| **Social** | *friends, going out, joining in, left behind* | **9** | 1 | 7 | 1 |
| **Everyday Activities** | *bathroom, morning routine, basic things* | **8** | 0 | 6 | 2 |
| **Pain/Physical Symptoms** | *dealing with pain, being in pain, respiratory* | **7** | 2 | 5 | 0 |
| **Time Management** | *balance, having enough time, trying to manage* | **6** | 1 | 4 | 1 |
| **Finding Care** | *personal care assistants* | **4** | 0 | 2 | 2 |
| **Mental Health** | *depression, feeling down, healthy mind* | **3** | 1 | 1 | 1 |
| **Self-Advocating** | *advocating, fight for myself* | **2** | 0 | 1 | 1 |
| **Other** | *Includes video game difficulty, unknown* | **3** | 1 | 1 | 1 |

**Additional file 1: Table S2. Key Words and Breakdown for Survey Question 2**

| **Question 2: How does SMA affect your schooling?** | | | | | |
| --- | --- | --- | --- | --- | --- |
| **Theme** | **Key Words** | **Total Responses** | **SMA Type** | | |
|  |  |  | **I** | **II** | **III** |
| **No Effect** | *it doesn’t, none, did not affect* | **13** | 0 | 8 | 5 |
| **Accessibility Accommodations** | *elevator, leaving early,* | **9** | 1 | 2 | 6 |
| **Aid and Assistance** | *need others assistance, 1 on 1 aide, para* | **9** | 2 | 7 | 0 |
| **Fatigue** | *tired easily, weak, lack of energy* | **8** | 1 | 6 | 1 |
| **Am Homeschooled/**  **Online School** | *home school, avoid germs* | **8** | 1 | 6 | 1 |
| **More Time and Effort** | *more difficult, extended time, more time to finish* | **8** | 0 | 8 | 0 |
| **Challenge to Keep Up** | *hard to make up, hard to keep up, missing school* | **7** | 1 | 5 | 1 |
| **Everyday Activities** | *bathroom, using locker, holding books, sports* | **7** | 0 | 5 | 2 |
| **Limited Participation** | *unable to participate, not involved with activities* | **7** | 0 | 5 | 2 |
| **Challenging to Write** | *writing, typing, pictures on IPad* | **4** | 0 | 2 | 2 |
| **Other** | *Includes pain, no friends, school distance* | **5** | 1 | 3 | 1 |

**Additional file 1: Table S3. Key Words and Breakdown for Survey Question 3**

| **Question 3: How does SMA affect your socialization?** | | | | | |
| --- | --- | --- | --- | --- | --- |
| **Theme** | **Key Words** | **Total Responses** | **SMA Type** | | |
|  |  |  | **I** | **II** | **III** |
| **No Effect** | *does not affect, little effect, it does not* | **16** | 2 | 8 | 6 |
| **Accessibility** | *limits locations, can’t go places* | **16** | 0 | 10 | 6 |
| **Activity Limitations** | *unable to participate, limits energy* | **14** | 3 | 10 | 1 |
| **Others Don't Understand** | *ableism, unable to relate, hard to understand* | **8** | 0 | 7 | 1 |
| **Dependence** | *need assistance, dependence on transportation and care* | **6** | 0 | 4 | 2 |
| **No Socialization** | *no friends, large effect* | **6** | 0 | 4 | 2 |
| **Communication Difficulties** | *avoid loud environments, hard to talk* | **6** | 1 | 5 | 0 |
| **Feeling Judged by Others** | *people judge me, others feel awkward* | **5** | 0 | 4 | 1 |
| **Dating** | *no girlfriend, dating impact* | **2** | 0 | 2 | 0 |
| **Other** | *inability to swallow, shy, overthinking relationships* | **6** | 0 | 3 | 3 |

**Additional file 1: Table S4. Key Words and Breakdown for Survey Question 4**

| **Question 4: What resources do you think should exist for teens and young adults with SMA? What do would you like to see created?** | | | | | |
| --- | --- | --- | --- | --- | --- |
| **Theme** | **Key Words** | **Total Responses** | **SMA Type** | | |
|  |  |  | **I** | **II** | **III** |
| **Support Groups** | *ways to connect, meet each other, support system* | **33** | 1 | 23 | 9 |
| **College/Independent Transition** | *college information, guidance towards independent living, transition program* | **11** | 1 | 8 | 2 |
| **Better Equipment/Technology** | *accessible cars, assistive technology, advanced wheelchairs* | **10** | 2 | 5 | 3 |
| **Unknown** | *I don’t know, no ideas* | **7** | 1 | 4 | 2 |
| **A Cure** | *Cure, cure for SMA* | **4** | 0 | 1 | 3 |
| **Big/Little Support Program** | *buddy system, pair individuals, buddy to learn from* | **4** | 0 | 4 | 0 |
| **Video Games** | *accessible gaming, gaming community* | **4** | 0 | 4 | 0 |
| **Accessible Activities** | *accessible waterpark, accessible pool, accessible sports* | **3** | 1 | 2 | 0 |
| **Accessible Transit Maps** | *accessibility transit maps, maps for accessible public transit* | **3** | 0 | 2 | 1 |
| **Caregivers and Funding** | *funding for personal care assistants, information on finding caregivers* | **3** | 0 | 2 | 1 |
| **Other** | *Includes I hope everything becomes simple, community cultural corridor* | **3** | 0 | 3 | 0 |
